# Four levers of reciprocity across human societies: concepts, analysis and predictions

**DOI:** 10.1017/ehs.2022.7

**Published:** 2022-02-21

**Authors:** Laurent Lehmann, Simon T. Powers, Carel P. van Schaik

**Affiliations:** 1 Department of Ecology and Evolution, University of Lausanne, Lausanne, Switzerland; 2 School of Computing, Edinburgh Napier University, Edinburgh, Scotland, UK; 3 Departments of Anthropology and Evolutionary Biology and Environmental Studies, and Center for the Interdisciplinary Study of Language Evolution, University of Zürich, Zürich, Switzerland

**Keywords:** Human evolution, large-scale societies, cooperation, reciprocity, rules, law

## Abstract

This paper surveys five human societal types – mobile foragers, horticulturalists, pre-state agriculturalists, state-based agriculturalists and liberal democracies – from the perspective of three core social problems faced by interacting individuals: coordination problems, social dilemmas and contest problems. We characterise the occurrence of these problems in the different societal types and enquire into the main force keeping societies together given the prevalence of these. To address this, we consider the social problems in light of the theory of repeated games, and delineate the role of intertemporal incentives in sustaining cooperative behaviour through the reciprocity principle. We analyse the population, economic and political structural features of the five societal types, and show that intertemporal incentives have been adapted to the changes in scope and scale of the core social problems as societies have grown in size. In all societies, reciprocity mechanisms appear to solve the social problems by enabling lifetime direct benefits to individuals for cooperation. Our analysis leads us to predict that as societies increase in complexity, they need more of the following four features to enable the scalability and adaptability of the reciprocity principle: nested grouping, decentralised enforcement and local information, centralised enforcement and coercive power, and formal rules.

## Graphical abstract

**Figure:**
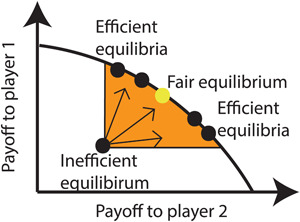

**Social media summary:** Four levers of reciprocity explain the scalability and adaptability of cooperation across human societies.

## 1. Introduction

From the mobile micro-bands filling the Pleistocene hunter–gatherer niche to the gigantic industrial states of the Anthropocene, human societies seem to display unlimited social scalability. The resources required to sustain small- and large-scale societies crucially rely on exchange and division of labour between individuals (Kaplan et al., 2009; Seabright, 2010). In turn, these activities rely on cooperative behaviour that needs to be maintained despite endless conflicts of interests. The defining feature of human societies is cooperation (Nolan & Lenski, 2014, p. 8, Raihani, 2021), and the occurrence of such behaviour needs to be explained. What, then, are the forces that can maintain cooperative social interactions in societies of arbitrary size? We address this question by conducting an analysis of the population, economic and political structural features across different societal types, based on two premises. The first premise concerns the human decision-making mechanism, which defines the constraints on behavioural decisions, and the second premise concerns the interaction mechanism (‘the rules of the game"), which specifies the constraints relating the behaviours of interacting individuals to their outcomes (Powers et al., 2021).

Following the logical primacy of evolutionary biology, our first premise is that individuals have evolved to express decision-making mechanisms that, over their lifetime, serve their genetic interests and thus treat interaction partners according to their degree of genetic relationship with them (Alexander, 1979, 1990, 2014). Genetic interests not only bond co-lateral relatives, but also provide a bridge between an individual alive today and their unborn relatives of the future, since natural selection can target an actor's phenotypic effects potentially up to hundreds of generations in the future (Lehmann, 2010; Lehmann & Rousset, 2012). On a proximate short-term level, this implies that individuals will tend to pursue material rewards for themselves and relatives, since this increases reproduction and survival. On a proximate medium-term level, this implies that individuals will pursue sources of material rewards, such as knowledge, reputation or influence, that correlate with power, which in turn correlates with reproduction and surviving enhancing resources. On a proximate long-term level, this implies that individuals will pursue states of the environment that correlate with the reproduction and survival of lineage members, possibly living far into the future. Genetic interests thus make for a complex motivational structure, whose crucial consequence is that individuals are not expected to behave systematically altruistically towards non-relatives, and thus not for the common good, unless they are incentivised to do so. In other words, natural selection tends to produce individuals who are (genetically) self-interested and incentive-abiding, a perspective that is essentially universally backed up by modelling work (Kay et al., 2020 for a review) and increasingly so in humans by behavioural experiments (Burton-Chellew & West, 2021 for a review).

Following the observation that humans face recurrent interaction situations, such as resource foraging, threat evasion, infrastructure building, teaching and learning, our second premise is that the defining feature of human social interactions is that they are repeated. Repeated interactions connect the present actions of an individual with their future consequences and so create incentives spanning different time periods, namely, intertemporal incentives, which allow for cooperation to be an equilibrium among self-interested individuals regardless of societal scale (e.g. Ostrom et al., 1994; Binmore, 2005, 2020; Mailath & Samuelson, 2006; Greif, 2006). Intertemporal incentives thus involve a trade-off between present and future payoffs, where present individual costs must in some way or another be balanced by future benefits that are contingent on the behaviour of the actor today. This mechanism of contingent cooperation is generically and colloquially referred to as the ‘reciprocity principle’ (Binmore, 1998, 2006, 2020).

It has been argued, mainly in the evolutionary human sciences, that the reciprocity principle applies realistically only to small groups or dyadic interactions, and that it is insufficient to explain the emergence of cooperation in large-scale societies, and therefore that new forces such as cultural-group selection are necessary (Boyd & Richerson, 1988; Fehr & Gächter, 2002; Richerson et al., 2016; Turchin, 2015). Yet it has also been emphasised that the reciprocity principle applies realistically regardless of group size and complexity, and so is sufficient to explain cooperation in societies of arbitrary size (Milgrom et al., 1990; Binmore, 2005; Powers et al., 2016; Stanish, 2017; Neumann, 2020). This is so because intertemporal incentives can be adapted by the individuals in a society and enabled through myriad different pathways: from encouraging children with various material and non-material rewards, ostracising group members by reputational ridicule or building a self-enforcing state having the ability to jail tax-evaders, to using crypto-currencies to elicit trustful transactions among anonymous renegades in self-policing decentralised digital networks. Human societal change can thus be retold as a story of the endogenous design of intertemporal incentives. While this perspective may appear self-evident to economists, it has been neglected in the evolutionary human sciences and we aim to develop it in this paper.

It is against the backdrop of these premises of individual behavioural self-interest and the repeated interactions they engage in that this paper analyses how the main subsistence types of societies, from (mobile) foraging to horticulturalist, agrarian and industrial societies (Nolan & Lenski, 2014, p. 64), constructed intertemporal incentives enabling cooperation. Using anthropological and macrosociological historical accounts (e.g. Johnson & Earle, 1987; Maryanski & Turner, 1993; Christian, 2004; Nolan & Lenski, 2014; Christian et al., 2014) along with results on repeated games (e.g. Fudenberg & Tirole, 1991; Osborne & Rubinstein, 1994; Aumann & Maschler, 1995; Mailath & Samuelson, 2006; Maschler et al., 2013), we carry out a conceptual analysis that consists of probing the consistency between the structural features of economic and political organisation and the conditions enabling the functioning of the reciprocity mechanism in complex societies. In other words, we take our data from history and structure our thinking with game theory and evolutionary biology, to delineate how intertemporal incentives have changed over time as a result of organisational change. The analysis of this paper refines and supports the hypothesis that all anatomically modern humans created institutional rules that transformed their interactions to allow self-interested individuals to remain prosocial as societies grew in size (Powers et al., 2016, 2021).

The rest of this paper is organised in four parts. First, in Section 2, we delineate a broad game-theoretic conceptual framework by: (a) defining social interactions and the core social problems of contest, coordination and social dilemma; (b) explaining how these problems can be solved by the reciprocity principle; and (c) identifying societal structural features that allow us to compare social problems and their solutions across different societies. Second, in Section 3, we use this framework to carry out an analysis explicitly comparing population, economic and political structural features of mobile foragers, horticulturalists, pre-state agrarian societies, state-based agrarian societies and liberal democracies. We document here both the occurrence of the social problems and how in each case the reciprocity principle is adapted to solve them. Third, in Section 4, we infer from the analysis that all complex societies use nested grouping, decentralised enforcement and local information, centralised enforcement and coercive power, and formal rules as the core levers of reciprocity, and delineate predictions pertaining to size-related relationships in human social organisation. Finally, in Section 5, we conclude with a number of open questions and avenues for future research.

## 2. Conceptual framework

### 2.1. Action situations and social problems

We consider a population of (genetically) self-interested individuals making up an autonomous group defining the scale of society (first premise). A society will generally be hierarchically clustered into groups (e.g. into clans and bands, into regions and villages or cities, into corporations and corporate units). We focus particular attention on the local group, which we take as the lowest level of grouping beyond the household at which most direct social interactions occur between individuals (e.g. the band, the village, the team, the department, the corporate unit). Our fundamental assumption is that individuals within groups, in particular local groups, face recurrent action situations (second premise). An action situation is defined as a set of specific behaviours and their associated outcomes that is mutually exclusive from another action situation (see the glossary in Table 1 for a more formal definition). For instance, resource foraging, mating, predator evasion, fire management and house-building can, in general, not be performed simultaneously and hence are by and large mutually exclusive, and so can all be taken as action situations.

**Table 1.** Glossary

Term	Description
Action situation	This consists of (a) a set of participating individuals, (b) a set of alternative feasible behaviours (action or stream of actions) to each such individual and (c) a transformation (mapping) from the behaviours of individuals to outcomes. This notion has been used as such in Ostrom et al. (1994) and it is equivalent to the notion of *game form* used in game theory (e.g. Fudenberg & Tirole, 1991; Osborne & Rubinstein, 1994; Hurwicz, 1996, p. 115) and the notion of *social interaction mechanism* used in Powers et al. (2021)
Efficiency	The standard definition of a group getting the most it can from its scarce resources (Mankiv, 2010, p. 5)
Social problem	Action situations whose outcome is likely to be inefficient
Excludability	Attribute of a resource whereby one person or group can easily exclude others from using that resource (e.g. Ostrom et al., 1994)
Rivalry	Attribute of a resource whereby one person or group consuming one unit of that resource makes it unavailable to others (e.g. Ostrom et al., 1994)
Private resource	Resource with high excludability, which makes it relatively straightforward to exclude potential beneficiaries within the local group (e.g. own resource gathered, own clothes). Such resources do not directly involve a social problem, but exchange of private resources as well as enforcement of private property rights involves social dilemmas
Public resource	Resource with low excludability and low rivalry, which makes it difficult to exclude potential beneficiaries, but whose consumption has low impact on others (e.g. defense, knowledge, street lightening, flood control). Non-excludability tends to create social dilemmas of production and depletion
Common-pool resource	Resources with low excludability and high rivalry, whose consumption does reduce availability to others (e.g. fish stock, game stock, local water). Such resources are the most prone to generate social problems
Direct reciprocity	Reciprocity mechanism where information to express contingent behaviour is gathered directly by the actors of the interaction
Indirect reciprocity	Reciprocity mechanism where information to express contingent behaviour is gathered indirectly by the actors through another party (e.g. Alexander, 1987; Ohtsuki and Iwasa, 2006)
Individual-to-individual reciprocity	Reciprocity mechanism where two or more individuals interact directly as autonomous agents (e.g. Alice interacts with Bob)
Individual-to-organisation reciprocity	Reciprocity mechanism where one ore more individuals interact with a structured collective of individuals that has its own identity (e.g. Alice interacts with a company or the the government)
Organisation-to-organisation reciprocity	Reciprocity mechanism where groups of individuals interact with each other (e.g. a company interacts with the government)

Interacting individuals in local groups necessarily encounter different action situations during their lifetime. Several if not all of them include outcomes that reduce welfare relative to other outcomes, e.g. wasted effort in time and energy, as well as outcomes that involve conflict and fighting between individuals and degradation of the environment. We generically refer to such action situations involving inefficient outcomes as social problems, and have identified three broad types of such problems.

(1) *Coordination problems.* Here, individuals face an action situation where they need to coordinate their action in the face of alternative equilibrium courses of action, which, if expressed by all individuals, no individual wishes others to change unilaterally. The social problem is that owing to the presence of alternative equilibria, some of them lead to more efficient outcomes, but individuals have difficulty coordinating to reach these outcomes (e.g. coordination games, stag-hunt games, social contract games, market games).

(2) *Social dilemmas.* Here, individuals produce and/or use and/or exchange a resource (or good or service or environmental effect), which results in either a beneficial outcome to group members (positive sum property) or a deleterious outcome (negative sum property). The social problem is that it is against an individual's immediate interest to exert effort to produce, refrain from consuming or exchange the resource (or information thereof), even though doing so would confer group welfare benefits and thus improve efficiency (e.g. prisoner's dilemma games, public goods games, tragedy of the commons games). There is thus a conflict between individual and collective interests, and the social problem is stronger than in a coordination problem, since outcomes obtain where all individuals wish others to change their behaviour. In the following, we will refer to the production, depletion and transaction social dilemmas in order to distinguish social problems pertaining to, respectively, the production, overuse and exchange of resources.

(3) *Contest problems.* Here, individuals compete for something they all want, but cannot all have. This zero-sum property of the interaction generates conflict between individuals. A key resource over which humans contest is reputation or status (e.g. hawk–dove games, mate choice games, games of contest). We also note that resources involved in contests can have opposing effects on the contestants (e.g. a tree between neighbours that one contestant wishes to clear and the other to keep).

On our reading of the literature, which spans the ranges of the social and evolutionary sciences, these three problems encapsulate broadly and concisely the types of situations that can lead to inefficient outcomes, and they have all been pointed out before in one way or another to be important (see, e.g. Olson, 1965; Hardin, 1968; Ullmann-Margalit, 1977; Skyrms, 1996; Kollock, 1998; Tullock, 2005; Binmore, 2005; Davies et al., 2012; Fukuyama, 2014; and in particular Schotter, 1981, p. 22; and Sugden, 1986, p. 149). It should also be emphasised that a given action situation may involve each of these three social problems. For instance, an irrigation system needs to be constructed and maintained (production social dilemma), the resource it carries is finite and needs to be shared (contest problem) and these problems may involve alternative courses of action that the group may take to solve them (coordination problem). Another example is that of exchange of scarce resources, which is the canonical problem addressed in economics (Smith, 1776 [1980]; Greif, 2000). At its base, this is a problem of coordination, but it also involves social dilemmas, in particular transaction dilemmas, since there is no guarantee that contractual obligations are upheld in the absence of external enforcement.

### 2.2. Social solutions through the reciprocity principle

We now recall that social problems can, in theory, all be solved by self-interested members of a society engaged in repeated and possibly stochastically varying action situations. Indeed, a fundamental, general and extensive set of game theory results that started to be developed in the 1950s, often colloquially referred to as the Folk Theorems, shows that whenever (a) individuals care sufficiently about future outcomes, (b) individuals have sufficient information about the past actions of co-players to hold them accountable and (c) the time horizons of interactions are unknown (death or termination of interactions is a random variable), then the efficient outcome of any action situation is available as equilibrium play in a repeated game (e.g. Luce & Raiffa, 1957; Fudenberg & Tirole, 1991; Myerson, 1991; Osborne & Rubinstein, 1994; Aumann & Maschler, 1995; Binmore, 1998, 2007, 2020; Mailath & Samuelson, 2006; Maschler et al., 2013). It is important to emphasise that this holds even if individuals have access to potentially incomplete information about partners’ actions, and even in non-dyadic interactions in groups of arbitrary size (e.g Fudenberg & Tirole, 1991; Binmore, 1998; Milgrom et al., 1990; Mailath & Samuelson, 2006; Maschler et al., 2013; Binmore, 2020). The link between present action and future outcomes creates intertemporal incentives that permit the endogeneous enforcement of commitments, agreements, contracts, pacts, property rights, production of public resources, refraining from depleting resources, fairness, trust and thus overall increase cooperation (see Box 1 for a more formal explanation of the logic of intertemporal incentives).

Box 1. **The logic of intertemporal incentives**

**Figure 1.**
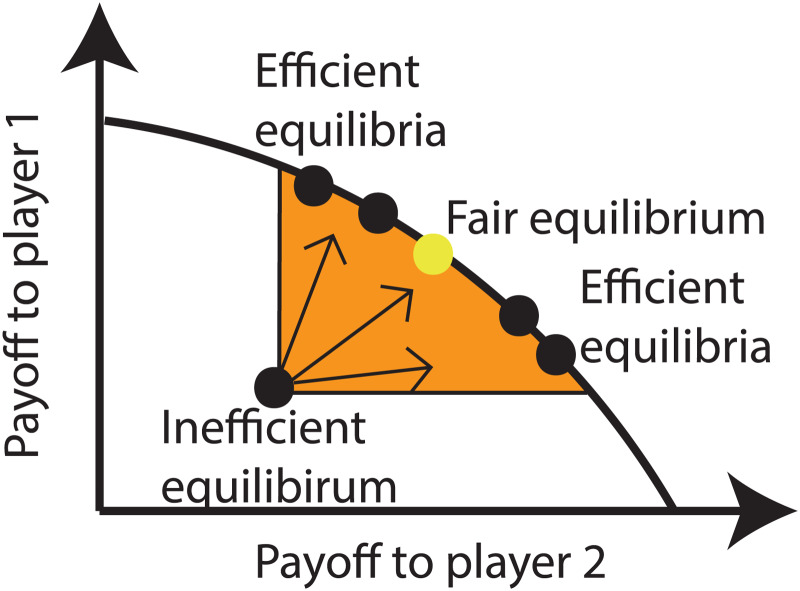
Folk Theorem for two-players' situation (adapted from Fig. 1 of Binmore (2014); we suggest Binmore (2007) and Seabright (1993) as accessible and lively accounts of the Folk theorem and Mailath and Samuelson (2006, pp. 33, 193, 232) for in-depth general treatments). The whole region (white and orange) below the curve joining the two axes represents the set of payoff pairs available to two players as the outcomes of a given action situation. The shaded region (orange) displays the pairs of average payoffs available as mixed-strategy Nash equilibria in the repeated version of the action situation (Nash equilibria of the game defined by eqn (1) of Box 1) and thus displays a multiplicity of equilibria available to the players that are compatible with the incentive structure of the action situation under focus. Three types of such Nash equilibria in this region are noteworthy. First, the inefficient equilibrium or reservation payoff to individuals obtained as the best then can do given others inflict the worst on them (i.e. the state of “war of all against all”). Second, the whole upper boundary of the orange region, which is a line of efficient equilibria where no individual can have its payoff increased without making the other player worse off. Finally, the fair equilibrium. Exactly the same logic applies mutatis mutandis to n-player interactions (see Box 1), with the only change being the dimensionality of the sets of the various equilibria. The Folk theorem says nothing about which equilibrium in the orange region obtains and this depends on how individuals organize the play of the action situation under focus. Interactions between a large number of unrelated and disorganized individuals is likely to result in the selection of an equilibrium close to the inefficient one: think of strangers attempting to construct a common irrigation system, but who lack any communication or coordination devices. As such, systems of group governance, where individuals structure their own interaction by way of devising rules of interactions - institutions - can favour more efficient equilibria (Ostrom, 1990; Gardner and Ostrom, 1991; Powers et al., 2016). This will push the system towards the efficient equilibrium region (arrows pointing upward). This region nonetheless still allows for very unequal resource distributions, but the present paper is not concerned with fairness or inequality issues (see Binmore, 1998, 2014 for considering such issues).

In order to understand how intertemporal incentives subtend cooperation, let's formalise the incentive structure of a single action situation, where the interaction is symmetrical for each player *i* ∈ {1, 2, …, *n*} among a set of *n* players, each having action set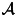. The expected material payoff *π*(*a*_*i*_, ***a***_−*i*_) to individual *i* depends on their own action, 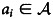, and the vector 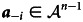 of actions of other players. An equilibrium of the interaction is characterised by a Nash equilibrium profile of actions, one for each individual, where no individual has an incentive to unilaterally change their action. In the standard prisoner's dilemma game with actions ‘cooperate’ C and ‘defect’ D (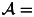
{C,D}), the Nash equilibrium means playing defect D. Suppose now that this action situation is indefinitely repeated. Then, the payoff to an individual can be written as
1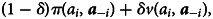 where *π*(*a*_*i*_, ***a***_−*i*_) is the present payoff, *v*(*a*_*i*_, ***a***_−*i*_)is the continuation payoff, formally the expectation of the *value function* of control theory, and *δ* weights the importance of future payoffs (e.g. any textbook on optimisation, control theory or repeated games, although in the present context Mailath &Samuelson, 2006, pp. 33, 193, 232 is a particularly valuable reference). When *δ* increases, the continuation payoff matters more as a behavioural incentive, and this payoff takes into account all possible future consequences (material or otherwise) of present actions and thus links present and future actions as an intertemporal trade-off. Equation (1) allows us to ascertain whether a unilateral deviation in action at present is profitable in the long term, which is decisive in the analysis of play of any repeated interaction (i.e. the ‘one shot deviation principle’, e.g. Mailath & Samuelson, 2006). For instance, eqn (1) shows that defecting in an *n*-player public goods game (increasing the present payoff, *π*) is not necessarily profitable, since it is likely to decrease *v*. In effect, other individuals will then likewise defect in the future or punish the individual, and if such defection or punishment occurs for a sufficiently large number of rounds, it will reduce the present incentives to defect. Yet calculating the Nash equilibrium of play becomes complicated since actions are intertemporally linked by way of individuals expressing *strategies*, which are conditional action expressions. This means that an untold number of different strategies can be shown to sustain cooperation in repeated interactions (this is the result of the Folk Theorem mentioned in Section 2.2 and Figure 1). Importantly, the logic of intertemporal incentives embodied in eqn (1) can in principle be extended to essentially any type of interaction, since what matters is that the current action *a*_*i*_ has some effect on the individual's continuation payoff *v*, and thus does not in itself hinge on any cognitive assumption: social microbes, plants and animals alike are subject to the trade-off encapsulated in eqn (1). As such, the logic of intertemporal incentives is stubbornly robust and broader than the classical game theory account based on rationality. Players with bounded rationality or with genetically determined strategies can even more easily cooperate under repeated interactions, for instance even in games with fixed finite time horizons (e.g. Neyman, 1985; McNamara et al., 2004; Shoham & Leyton-Brown, 2009, pp. 150–153), and the Folk Theorem result (Figure 1) can be sustained by simple neural networks (Cho, 1994). Further, many models in evolutionary biology not overtly using the language of intertemporal incentives are precisely built on this principle (e.g. McNamara et al., 1999; Eshel & Shaked, 2001; Roberts, 2005), so these models should not be thought of as alternative pathways to cooperation. Some work in evolutionary biology does use the concept of reciprocity broadly (e.g. Trivers, 1971; Alexander, 1987; Lehmann & Keller, 2006; Akçay & Cleve, 2014; Carter, 2014). Nevertheless, there remain open theoretical questions concerning the generality of the Folk Theorem itself for certain types of private information structures (Kandori & Obara, 2006).

Following Binmore (1998, 2006, 2020), we refer to the general finding that cooperation can be maintained by intertemporal incentives through individuals expressing contingent behaviour as the *reciprocity principle*. Since intertemporal incentives can be enforced by very different means and strategies, it is useful to distinguish between various forms of mechanisms of reciprocity (see the glossary in Table 1 and Section 3). Importantly, the reciprocity principle entails not only that efficient outcomes can obtain, but also that any outcome of a one-shot action situation on which players might agree if they could write enforceable contracts is available as an equilibrium of the associated repeated game (Binmore, 2005, p. 81, 2020, p. 87; Mailath & Samuelson, 2006, chapter 5.7). A multiplicity of outcomes is thus available to players when action situations are repeated. The reciprocity principle is therefore an abstract result and in itself cannot predict the actual type of behaviour that will be expressed in any particular action situation and whether an efficient equilibrium will actually be played. What it crucially demonstrates is that (a) cooperation is feasible regardless of societal scale and (b) the fundamental problem faced by individuals in a society is a selection problem among the myriad possible alternative ways of structuring their interactions and expressing equilibrium behaviours. In other words, the reciprocity principle asserts that both contest problems and social dilemmas can be transformed into coordination problems, which thus become the main problem to solve in a society (see Figure 1).

### Political and economic structural features

Because humans can actively communicate and recognise their collective coordination needs, they attempt to deliberately structure their interactions to some extent at least (Fukuyama, 2011, p. 446). It is often emphasised that more formal interactions tend to be more deliberately planned, while less formal ones more spontaneously ordered (e.g. Schotter, 1981; North, 1990, 1991; Ostrom et al., 1994; Aoki, 2001; Greif, 2006; Brousseau et al., 2011). It is thus useful to recognise that individuals in human societies are engaged in two qualitatively different types of action situation: (a) playing economically relevant action situations, whose outcomes determine welfare or material payoff; and (b) the active genesis of the rules of (repeated) economic action situations through communication and bargaining by all or a subset of group members. Hence, by attempting to promote social orders suiting their needs, individuals are engaged in both economic and political action situations, which is the hallmark of institutionalised rule determination (Hurwicz, 1996; Brousseau & Raynaud, 2011; Powers et al., 2016).

The defining feature of political interactions is thus a group decision-making process over alternative modes of interactions, and hence a way of affecting equilibrium play of payoff-relevant economic interactions. Regardless of the mechanistic details of this complicated process, there are many ways of changing the rules of action situations to influence behavioural expression (see Box 2). In order to identify and compare the prevalence of social problems and their solutions across different societies, we need a conceptualisation of the key structural features of a society pertaining to cooperative interactions. It follows directly from the game theoretic framework reviewed above that one should at least focus on the three following broad types of structural features.

(1) *Population structure.* This describes number of individuals in action situations, and features pertaining to their physical distribution and connection to others in a society. Here, it is relevant to consider how individuals are distributed spatially within and between groups, as well as the size of groups and the type of interaction networks.

(2) *Economic structure.* This describes societal features pertaining to economically relevant action situations, namely, to the mode of subsistence and the main factors of production thereof, such as labour and infrastructures used in resource acquisition. Here, it is relevant to consider resource distribution factors, such as type of resource ownership (private or collective property) and how resources and goods are re-distributed or exchanged in the society. Since the mode of subsistence may involve the appropriation of resources produced by others through raiding and warfare, it is also relevant to consider factors of appropriation and their correlates, such as group defence.

(3) *Political structure.* This describes societal features pertaining to politically relevant action situations, namely, to its organisation as a collective identity and its capacity at self-governance, as well as the ability to make and enforce rules. Here, it is relevant to consider features of group decision-making processes, such as how and by whom decisions affecting rules are taken, e.g. whether the process is centralised or decentralised, or to what extent power is delegated. Finally, it is relevant to consider factors of *enforcement* of group decisions such as coercive capacity.

In focusing on these structural features, we are clearly and voluntarily omitting a number of dimensions that have been deemed relevant for describing a human society (e.g. Maryanski & Turner, 1993; Nolan & Lenski, 2014; van Schaik, 2016). However, these three structural features have been generally recognised as being part of the most basic components of human societies (Nolan & Lenski, 2014, pp. 25–43), they are operational, and crucially they allow us to compare reciprocity mechanisms across societies.

Box 2. **Intertemporal incentive transformation**

In order to recognise the many ways in which behavioural constraints can be endogenously changed, it is useful to consider a more extensive description of an action situation, which, according to the Glossary in Table 1, includes (a) a set of individuals, (b) a set of alternative behaviours available to each individual and (c) a transformation from behavioural profiles in the group to outcomes. The latter element itself involves at least three relevant elements (e.g. Gardner & Ostrom, 1991; Ostrom et al., 1994 or more generally any game theory textbook): (a) attributes for each individual characterising its state in the interaction, such as age or role as leader or subordinate, and the information available to them; (b) the exogenous and endogenous environmental state; and (c) consequence rules that map behavioural decisions in given environmental states to intermediate or final outcomes. These consequence rules are the outcomes of (possibly sequential) interactions between individuals as well as transformations of different material inputs into outputs (e.g. extracting resources, exchanging them, transforming them to new resources and goods) that eventually lead to payoff-relevant outcomes. Let us now give some examples of how the structural features of action situations can be changed in the context of the reciprocity principle (see also Gardner & Ostrom, 1991; Ostrom et al., 1994 for many concrete examples across several domains, and Powers et al., 2021 in the context of cooperation in large-scale societies). For instance, more or fewer individuals can be included into an action situation. Reducing the number of individuals increases the working of the reciprocity mechanism, since information about the behaviour of others is more reliable. In large-scale societies, this can be achieved by making interactions occur in small groups, as when they occur among friends, among the members of a team, or any coalition of individuals. Any technology that increases the flow and accuracy of information about past behaviour, by making behaviour more visible, public or recorded, will increase the effectiveness of the reciprocity mechanism. Behavioural options and strategies can also be restricted or expanded. For instance, a certain behaviour can be outlawed or favoured and this can further be affected by various modes of transmission, from teaching prosocial behaviour to ostracising those not expressing it. These features will all facilitate the working of the reciprocity mechanism. In order to avoid behavioural decisions needing to be taken on each repetition of an action situation, regularity of behaviour can be suggested or enforced in the form of agreements, contracts or norms. Finally, it is worth mentioning that the field of *mechanism design* in game theory is specifically devoted to formally determining rules of interactions in order to achieve specific societal outcomes (e.g. Groves et al., 1987; Fudenberg & Tirole, 1991, chapter 7; Osborne & Rubinstein, 1994, chapter 10; Narahari, 2014), and has produced many concrete applications (e.g. Binmore & Klemperer, 2002; Tirole, 2017). Hence, while the first mechanism designers, the mobile foragers, used intuition to structure their interactions, modern societies use an armada of formally trained scientists to attempt to do so.

## 3. Social problems and their solutions across societal types

We now use the concepts introduced in Section 2 to delineate the type of social problems encountered by different types of societies, and analyse their solution by way of adapting the functioning of the reciprocity principle. To carry out this analysis, we need a taxonomy of societies. Here, we follow Nolan and Lenski (2014) by distinguishing societies in terms of their primary mode of subsistence. Not only does this approach mirror that of animal socioecology (van Schaik, 2016), but this taxonomy is particularly useful in our context because it directly maps onto the economic structural features delineated in Section 2.3. This then allows for a straightforward identification of the social problems, since different resource types used for subsistence are directly linked to the occurrence of social problems (see the Glossary in Table 1).

We consider five types of societies: mobile foragers, horticulturalists, pre-state agriculturalists, state-based agriculturalists and industrial societies in the form of liberal democracies. While these cover the major transitions in societal types (Nolan & Lenski, 2014, p. 66), we are omitting some well-defined subsistence types, such as sedentary foragers, because they are not on the main line of transition to industrial societies. Also, among the different types of industrial societies, we choose to focus on liberal democracies since their partly decentralised governments and massive scales of free trade make them, *a priori*, most prone to social problems.

In order to be able to identify the social problems and their solutions for each societal type we proceed as follows. First, we identify the societal structural features (Section 2.3) and these are summarised for each society in the Supplementary Material in Tables S1–S5, whose construction is explained in this Supplementary Material. From these tables and using the standard classification of resources into private, public and common-pool resources (see the Glossary) we identify the prevalence of the three core social problems (recall Section 2.1). Then, using the convenient classification of reciprocity mechanisms into direct and indirect reciprocity, as well as into individual-to-individual, individual-to-organisation and organisation-to-organisation reciprocity (see Glossary), we discuss the implementation of the reciprocity principle in each society.

### 3.1. Mobile foragers

From Supplementary Material Table S1, we infer that mobile foragers face all three social problems. Indeed, owing to their intrinsic group-living mode and the abundant use of resources collected communally and shared, coordination problems and social dilemmas necessarily occur in mobile foragers, most notably concerning meat hunting and exchange (Kelly, 2013; Marlowe, 2005). Since these resources are further mostly of the common-pool resource type, contest problems must also occur. At the same time, mobile foragers have essentially no infrastructure and no or few common-pool resources are produced endogenously. Further, no real depletion problems occur, since groups are mobile and move to find new resources when foraging yields decline (depletion may occur at the population level). Hence, even if mobile foragers face all social problems, their scales remain moderate.

How are these social problems solved? Owing to small local group size, there is essentially complete or perfect information diffusion within groups. Behaviour is visible and can be monitored, and there is no clear distinction between private and public life. Further, individuals are together for long stretches of time that total up to years, if not their whole lifespan. All of this entails behavioural interdependence and allows for strong intertemporal incentives to build up, which are potentially affected by all local group members. This implies that social dilemmas and contest problems can be solved by direct and indirect individual-to-individual reciprocity as well as individual-to-organisation reciprocity, for instance by implementing sanctions in a coalitionary way (Boehm, 1999). Coordination problems impacting the whole local group can then be solved by having assemblies of individuals negotiating courses of action through consensus building, which, in turn, can be locally enforced in a coalitionary way. This process allows for creating and changing the rules of interaction such as who should give meat to others (Testart, 1987; Kaplan et al., 2005, 2009). There are also occasional meetings of bands with many individuals present and some decisions impacting the community are taken, such are rules for marriage and perhaps involvement in warfare (Layton et al., 2012).

### 3.2. Horticulturalists

From Supplementary Material Table S2, we infer that the scale of the three social problems increases among horticulturalists relative to that in mobile foragers. Not only are the three problems prevalent owing to the communal mode of living and the use of common-pool resources for subsistence, but there is also an increase in these problems associated with the more sedentary mode of living and the higher reliance on planting and gardening. Communal houses need to be built, and gardens and irrigation systems need to be managed. More activities thus require coordination among group members and more activities involve social dilemmas and contest problems. In particular, depletion problems at the local scale can occur, since competition for local resources such as water and soil increases. Further, there is an increase in raiding between groups, which requires defensive coalitions to be ready at all times.

These social problems can still be solved in a straightforward way. Indeed, owing to the reasonably small local group size, there is still much information diffusion between group members and no clear distinction between private and public life, much like in mobile forager groups. An individual not pulling their weight in any action situation can easily be detected, which creates credible intertemporal incentives for cooperation. Overall, there are thus no more fundamental threats to the implementation of the reciprocity mechanism at the local scale in horticulturalists than in mobile foragers. Cooperation can be maintained largely by direct and indirect individual-to-individual and individual-to-organisation reciprocity in all action situations. Yet the increase in coordination problems pertaining to group activity, especially in areas of higher density, results in more centralised group-decision mechanisms than consensus building. In horticulturalists, we see the appearance of local leadership such as ‘Big men’ that start to have some capacity for coercion (Earle, 1997; Flannery & Marcus, 2014). In advanced horticulturalists they gain the ability to collect taxes from group members, where these resources can be partly allocated to monitoring and enforcement processes, thus improving the functioning of the reciprocity mechanism (Flannery & Marcus, 2014).

### 3.3. Pre-state agriculturalists

From Supplementary Material Table S3, we infer a quantitative increase in all three social problems faced by pre-state agriculturalists relative to mobile foraging and horticulturalist societies. First, the greater division of labour and specialisation requires more coordination. Second, the increase in infrastructures entails that more common-pool resources are produced endogenously, which increases the scope of social dilemmas (yet ‘wheat’ and ‘rice’ types of agriculturalist markedly differ in the amount of capital or infrastructure needed). Third, the increase in population density and permanency of settlement may impact soil and water to the point of local depletion. All of this increases social problems and implies that the behaviour of others becomes more difficult to monitor, since the lack of communal housing and larger densities make it no longer possible to remember all faces, favours and facts from group members. The result is more incomplete information.

How then are the social problems solved? In pre-state agriculturalists societies, we see the appearance of two connected mechanisms for that. First, there is the appearance of novel rules and technology of interactions, in particular the private property of resources and their exchange by way of emerging monetary systems (Nolan & Lenski, 2014). Private property links the present and future state of a resource and creates intertemporal incentives about its production at the level of the individual, and that of its descendent lineage through inheritance. This simply eliminates social problems and so the reciprocity principle is not even needed at the private scale (yet enforcement and exchange of private resources involve social problems). Money, which serves three functions – storage of value, medium of exchange and unit of account (Mankiv, 2010) – has been used under many forms such as shells, furs and grain (Szabo, 2002; Nolan & Lenski, 2014). It solves the fundamental problem of the *double coincidence of wants*, which is the unlikely occurrence that two people each have a resource the other wants (Mankiv, 2010, p. 220). By using a medium of exchange and more generally money, there is no longer any need to search and track who has what, who owned what and what was exchanged in the past. This dramatically reduces transaction costs and enables the functioning of individual-to-individual reciprocity in more diffuse groups of interacting individuals (Szabo, 2002).

Second, in pre-state agriculturalists we see the appearance of leadership with centralised coercive capacity, which is based on ownership of land and other forms of wealth allowing influence to be bought. Owing to the capacity of coerciveness, group decisions can be implemented and social dilemmas and contest problems diffused by way of individual-to-organisation reciprocity. It is important to note that while delegating power to leadership effectively solves coordination problems (Calvert, 1992), it also paves the way for new social dilemmas. Indeed, the increasing surrender of power to the leadership can benefit leaders as much if not more than it benefits the group, since leaders themselves are self-interested. This opens the door to the politician's dilemma (Geddes, 1994; Tullock, 2005) and shows that a deliberative change of social structure that involves a shift from a negotiated social order (as observed in mobile foragers and horticulturalist) to a more mandatory one can end up decreasing group efficiency.

### 3.4. State-based agriculturalists

From Supplementary Material Table S4, we infer in archaic states a continuation of the trend of an increase in all three social problems associated with sedentarisation and surplus production. First, there is a further increase in the division of labour and specialisation, which increases coordination needs. Second, there is an increase in the collective infrastructures of production as well as defensive infrastructures owing to the virtually continuous presence of warfare (e.g. walled cities; Gat, 2006), which increases the scope for social dilemmas. Thirdly, many resources are of the common-pool type and can be locally depleted, e.g. deforestation and water salinisation (Scott, 2017), and this creates more contest problems. In parallel, the advent of cities implies that anonymity markedly increases among individuals living in the same spatial area. How then are the increasing number of social problems solved in archaic states?

At the level of production and exchange of private resources, there is an increase in the use of money that becomes fully integrated into the economy (Christian et al., 2014; Nolan & Lenski, 2014) and allows multiplayer interactions to be made effectively dyadic (Shubik, 1987). This reduces the need for trust and enables the working of a fully decentralised price mechanism, which allows the efficient coordination of actions and brings benefits to individuals from knowledge they do not have (Hayek, 1945; Mount & Reiter, 1974; Hammond, 1979). We also see the appearance of private order institutions to solve exchange dilemmas, whose order-creating mechanism is to use reputation to link past behaviour and the future payoff of individuals, thus generating intertemporal incentives through indirect reciprocity (Milgrom et al., 1990; Clay, 1997). With respect to the production of common-pool and public resources, in villages, where most primary production takes place, these resources can be managed by the formation of self-policing communities that enforce the maintenance of cooperation by individual-to-organisation reciprocity and active communication between group members (Ostrom, 1990; Milgrom et al., 1990; Gardner & Ostrom, 1991; Ostrom et al., 1994; Greif et al., 1994; Greif, 2006). These corporate structures involve local villages of farmers as well as market towns and guilds (Christian et al., 2014). They are often based on polycentric governance with multiple centres of (collective) decision making, each of which consists of sizeable groups of individuals operating with some degree of autonomy and that organise themselves according to specific ends (Ostrom, 2010; Carlisle & Gruby, 2017). This enables the implementation of the reciprocity mechanism within and between groups. These changes in organisational structure also imply that the local group of an individual is no longer tied to spatial proximity, but depends on the action situation, each of which may involve a different interaction network and hence ‘local group’.

The production of common-pool or public resources can also be enforced by higher governance levels, since the state now has the monopoly on coercion and itself uses corporate entities to organise public service. If the bureaucracy and the army are well organised, then large-scale projects can be achieved (e.g. construction of pyramids) that are enforced through religious, legal and physical threats that all create and maintain intertemporal incentives. The state obtains resources through heavy taxation of primary production, but does not interfere much otherwise and so the system of production remains partly decentralised (Christian, 2004). Finally, in archaic states, we also see the appearance of more explicitly codified legal rules, elicited by the appearance of technology such as writing and calendars, which permit the tracking of obligations and stable rules of organisation. Overall, social problems can be solved and there is no real threat to the working of the reciprocity mechanism.

### 3.5. Liberal democracies

From Supplementary Material Table S5, we infer that in modern liberal democracies social problems reach an unparalleled level. The extreme division of labour and power, where labour is increasingly delegated to machines and power is increasingly delegated to the legislature, requires coordination at all level of social organisation. It is difficult to overstate the need for coordination in a society where all individuals are completely interdependent and rely on others for essentially all their needs (Seabright, 2010). The massive public infrastructures for transportation, schooling, research and defence all involve the production of common-pool and public resources, which opens the door for multiple social dilemmas and these dilemmas also permeate all level of organisation. Similarly, contest problems are manifold owing to the many resources that can be used to the point of depletion on a planetary scale. In other words, there is scope for social problems all over the place in large-scale societies.

These social problems are solved by different means. Coordination problems linked to the private exchange of resources are solved by modern decentralised markets (Pindyck & Rubinfeld, 2001; Mankiv, 2010). Owing to the advent of information technology, these can be implemented on a worldwide scale and display unlimited social scalability since digital information is non-rivalrous. One example of a technological breakthrough is the blockchain, which is a decentralised public database containing the immutable shared history of information it was designed to store (Balachandran, 2020). This enables the implementation of indirect reciprocity even among complete strangers interacting only via remote sensing technology. Various rules of organisation and technological advances thus bolster the functioning of individual-to-individual and individual-to-organisation reciprocity in a manner that is not unlike that in mobile foragers, since society is structured in a variety of networks of interactions that replace the ‘band’, but where individuals no longer share the same spatial location. For individuals that do, social organisation can be structured to meet the mobile forager's band level of monitoring of others. A particularly salient example is Japan, where the structuring of schools, offices within corporations, company housing, family houses and neighbourhoods means that Japanese live under almost constant supervision by group members, which makes their behaviour particularly highly visible to others and thus accountable (Hechter & Kanazawa, 1993). Coordination problems linked to firm or state organisation are solved by vertical integration and bureaucratic control, all of which are facilitated by information technology, which tends to augment common knowledge and so improves coordination. Corporations themselves become the predominant social structure linked to resource production, as they allow the reduction of the transaction costs inherent in production and exchange (Coase, 1937, 1960). This makes it possible to enforce the reciprocity mechanism at the corporate level and thus allows for organisation-to-organisation reciprocity (also underlying the formation of cartels).

In liberal democracies, the state retains its capacity to extract revenues. It also has nearly unlimited legal capacity of enforcement and a monopoly on the use of force, without which capital and assets are unlikely to exist (Pastor, 2019, p. (19). The accountability of the liberal state (Fukuyama, 2011, 2014) limits contests within as well as between states (Gat, 2017), thus better solving the social problems of bureaucracy and reducing the politician's dilemma. This can lead to an open-ended virtuous loop (Brousseau et al., 2010). The state is able to guarantee security and the proper enforcement of contracts and property rights, thus securing the reciprocity principle. The efficient economy enables the government to derive increased benefits. In turn, demands for common-pool and public resources underlying resource production are increasingly better used and addressed as the benefits from economies of scale (brought by size) and scope (brought by diversity, e.g. Panzar & Willig, 1981) are better exploited on the economic side, which itself hinges on improvements in the reciprocity mechanism brought by the legal and technological side. In other words, among the myriad equilibria of the interactions, more efficient ones can be reached when there is a feedback between economic rule creation, enforcement and accountability, all of which hinge on intertemporal incentives. This also implies that the state now has a prodigious ability to regulate its citizens’ daily lives by using legislation to enable coercion. As such, behaviour becomes more confined to a tight window of legal and administrative rules and some of these rules can generate social problems where none existed before, for instance when legal privileges are created like patents on knowledge commons (Pastor, 2019) or when vetocracy makes change ineffective (Fukuyama, 2014).

## 4. Fours levers of reciprocity and predictions

The analysis of the previous section shows that all human societies face the three core social problems and that societal structural features reflect the continuing functioning of the reciprocity mechanism to diffuse these problems. The underlying processes can be more or less formal, more or less apparent to the interacting individuals, and be implemented by different means. This reflects the wide variety of social organisations that may be history dependent. Yet our comparative analysis points to the following four broad levers of reciprocity.

(1) *Nested grouping.* Grouping individuals into lower level units, e.g. bands within communities or clans within tribes, or teams within factories and factories within companies, brings two separate advantages. First, it decreases the effective number of players in the interaction, which makes behaviour more visible and enforceable. Second, it allows whole groups to function as players of repeated games against other groups. This increases the functioning of individual-to-individual reciprocity within groups and organisation-to-organisation reciprocity between groups.

(2) *Decentralised enforcement and local information.* Decentralised enforcement, done by individuals using local information directly relevant to their private resources and interactions, brings the advantage of avoiding needing to amalgamate dispersed and fragmented information at the group level. This reduces information costs where division of labour and anonymity make the monitoring of behaviour more partial and incomplete. Local information and privacy make indirect reciprocity more relevant, and its functioning increases with the level of technology to access information about others’ behaviour (e.g. information networks, writing systems, money).

(3) *Centralised enforcement and coercive power.* A central authority with the monopoly of coercion brings the advantage of being able to enforce coordination and cooperation at essentially all levels of organisation. Coercive power, which is coalitionary in nature, can obtain in small-scale societies and allows the transformation of multiplayer game situations into a two player game: a focal actor on one side, which could be a single individual or a unitary group, and on the other side the centralised coercive (coalitionary) power. This increases the functioning of the reciprocity mechanism within groups by individual-to-organisation as well as organisation-to-organisation reciprocity.

(4) *Rules.* Rules, whether informal or formal, bring the advantage of isolating equilibria and patterns of outcomes among alternatives. They reduce search and transactions costs as well as uncertainty, which is especially useful in large societies where diversity between individuals will lead to proportionally more contest problems (since the total number of pairs of possible conflictual situations relative to group size increases with group size). Further, the implementation of the three previous levers of reciprocity, nested grouping, centralised enforcement and decentralised enforcement, all depend on endogenously devised rules of social organisation that, for any given action situation, can increase the functioning of the reciprocity principle (recall Figure 1).

These four levers of reciprocity can be regarded as societal-wide traits, which leads us to make three points about them. First, comparison between the different societal types (Section 3 and Supplementary Material Tables S1–S5) shows that all societies have them at least at the local group level, and all complex societies at the societal level (see Table 2). In other words, in all societies, even mobile foragers, there is an attempt to control individual behaviour by having politically determined deterrence mechanisms based on coalitionary power that are super-imposed on individual day-to-day interactions. This is consistent with the view that the context of cooperation did not change qualitatively as societies expanded in size (Powers et al., 2021). Quantitatively, however, one can infer by comparing societal types (Section 3 and Supplementary Material Tables S1–S5) that the prevalence of the levers of reciprocity has changed over time. In particular, we make the following predictions: (a) the larger a society, the more levels of nested grouping it has and the more important centralised coercive power becomes in solving the society- wide social problems; (b) the more privacy and division of labour in a society, the more indirect reciprocity (as opposed to direct reciprocity) solves coordination and exchange dilemmas; and (c) the more societal diversity there is, the more rules are needed to address them.

**Table 2.** Reciprocity levers across societal types

Cases	Nested grouping	Decentralised enforcement	Centralised enforcement (local group)	Centralised enforcement (societal level)	Informal rules	Formal rules
Mobile foragers	✓	✓	✓	✗	✓	✗
Horticulturalists	✓	✓	✓	✗	✓	✗
Pre-state agriculturalists	✓	✓	✓	✗	✓	✗
State based agriculturalists	✓	✓	✓	✓	✓	✓
Liberal democracies	✓	✓	✓	✓	✓	✓

A check mark represents the presence of a given lever of reciprocity in a given societal type (obtained by comparing societal types in Section 3 and Supplementary Material Tables S1–S5). We divided the lever of centralised enforcement into whether it obtains at the local group level or the society at large; in the latter case it must involve the monopoly of coercion at the societal level. We also divided the lever of rules into informal rules, which are passed down by tradition, and formal rules that have some form of physical repository (outside the minds of individuals), most notably written rules.

Second, the intertemporal incentives in large-scale societies will generally depend on the joint occurrence of all four levers of reciprocity. For instance, large-scale social dilemmas cannot be contained without centralised coercive power, since even in the implausible situation where all common-pool resources needed for survival and reproduction were privatised, only centralised power is able to promulgate binding rules (see Pastor, 2019 for a strong emphasis on this point). Enforcement of these rules, whether delegated to a bureaucracy or a private entity, cannot be enabled without a well-functioning coalitionary entity. This relies on nested grouping of its component parts, and rules regulating its interactions with the economic world, whose efficiency in providing resources and goods is highly dependent on decentralised exchange networks. Thus, the levers of reciprocity feed back on each other. This makes our hypothesis that intertemporal incentives are at the nexus of human cooperation a composite one, since it considers predictions of societal organisation from the joint perspective of the four levers of reciprocity. Thus, if a society larger than pre-state agriculturalists with high division of labour converges on an efficient cooperative equilibrium, but lacks any one of the four levers, this would suggest either that mechanisms other than reciprocity operate to solve the social problems, or that a single lever of reciprocity swamps all others. In both cases, this would refute our perspective. An example would be a large-scale, market-based society, extracting and manufacturing all of its resources (‘autarkic society’) without any centralised coercive power, and thus relying exclusively on individual-to-individual indirect reciprocity to enable cooperation.

Third, we note that all levers of reciprocity have in one way or another been stressed before to be important to social organisation in both the social and evolutionary sciences, e.g. respectively from the two fields, Ostrom et al. (1994) and Bourke (2011) for nested grouping; Weber (1946) and Ågren et al. (2019) for centralised enforcement and coercive power (sometimes also called the single power principle, Barnett, 2014); Hayek (1945) and Alexander (1987) for decentralised enforcement and local information; and Brennan and Buchanan (1985) and Singh et al. (2017) for rules. However, here we have considered these traits from the overarching perspective of the scaffolding of intertemporal incentives. As such, our prediction is that all levers of reciprocity have been shaped by societal socio-cultural evolution for that functional role to some extent at least.

## 5. Discussion

We have delineated a conceptual framework based on evolutionary biology and game theory orthodoxy (Section 2) and used it to analyse how continually arising and changing social problems are persistently solved in humans societies by adapting the reciprocity principle (Section 3), in particular, the proper functioning of monitoring and enforcement mechanisms that enable lifetime direct benefits to individuals for cooperation in societies regardless of scale. This is in line with the institutional-path hypothesis for the emergence of large-scale societies (Powers et al., 2016, 2021). Our analysis also suggests that all complex societies solve the social problems by attuning the four levers of reciprocity, namely, nested grouping, decentralised enforcement and local information, centralised enforcement and coercive power, and rules (Section 4). We conjecture that these four levers of reciprocity are necessary and sufficient to enable the scalability of cooperation in human groups of arbitrary size.

We have left out from our analysis a number of factors that have been deemed important for understanding human cooperation such as, for instance, trust and rituals (Fukuyama, 1995; Stanish, 2017). Trust increases group cohesion and relies on shared informal rules subordinating individual to common interests (Fukuyama, 1995). However, such rules can be equilibria of repeated interactions when behaviour is incentive-driven only when the reciprocity mechanism is functioning properly. Rituals have been proposed to establish rules for social organisation and act as group coordination devices in the absence of money in pre-state societies (Stanish, 2017). Here too, rituals are behavioural equilibria that should be understood in game-theoretic terms, and here it is acknowledged that they operate under the scaffold of the reciprocity principle (Stanish, 2017, p. 80). As such, we do not deny that cultural factors can matter for cooperation, but regard them as reflecting self-sustaining patterns of beliefs, motives and behaviours that arise as equilibria under multilevel repeated interaction settings (see Powers et al., 2021 for a discussion of the compatibility of this perspective with the cultural group selection hypothesis). It is in fact one of the fundamental strengths of the reciprocity principle that it is able to accommodate much cultural variation in the organisational features observed in different times and places (Mailath & Samuelson, 2006). By the same token, we have left out considering explicitly the various partner control mechanisms of decentralised enforcement (Bshary & Bronstein, 2011), such as partner choice, partner switching or sanctioning, as well as the various social norms underlying reputation dynamics (Ohtsuki & Iwasa, 2006, 2007). Here too, control, communication and assessment mechanisms matter at the mechanistic behavioural level, but, again, the reciprocity principle makes eligible much variation in behaviour.

The four levers of reciprocity should thus be regarded as coarse-grained factors shaping inter-temporal incentives that can be collectively modified by interacting individuals. In other words, the four levers of reciprocity should be regarded as group-wide traits. This leads directly to a cultural evolutionary perspective on their change and paves the way for two lines of future research. First, cross-cultural comparative studies of socio-political and economic organisation (e.g. Currie et al., 2010; Currie & Mace, 2011; Sheehan et al., 2018) could be undertaken in order to determine the relationship between the presence or absence of social problems and the presence or absence of any of the levers of reciprocity. This should help reconstruct their evolutionary history, ascertain their functional role as well as determine the causal co-evolutionary pathways to social organisation. Second, formal models of socio-political and economic co-evolution could be devised. So far, the bulk of evolutionary game theory modelling has been devoted to studying the emergence of cooperation under specific economic action situations. Much less modelling has been concerned with political change, and integrating both layers of interactions as a co-evolutionary process is a fundamental challenge for future interdisciplinary modelling work (Currie et al., 2021, for some attempts in this direction see e.g. Powers & Lehmann, 2013; Frey & Sumner, 2019; Frey & Atkisson, 2020; Perret et al., 2020; Currie et al., 2021). More generally, a number of outstanding questions remain about the emergence of intertemporal incentives in complex human societies, when regarded as outcomes of a conflictual political process that is cultural evolutionary in nature (see Box 3).

Box 3. **Outstanding questions**

We here delineate a number of open question concerning the emergence and maintenance of cooperation in large-scale societies.

- What is the optimal combination between centralised and decentralised enforcement to implement the reciprocity principle in given action situations in large-scale societies?
- To what extent can innovations in information technology alone help solve social problems through facilitating the use of information and the functioning of indirect reciprocity?
- What is the appropriate medium of coercion in social dilemmas from the perspective of benefits to group welfare, or can coercion be enforced without any punishment (e.g. Barnett, 2014)?
- How does the number of formal rules of social organisation change with societal size, e.g. do the number of rules increase more than linearly with size?
- Is there a specific relationship between levers of reciprocity and social problems, so that acting on a given lever is more appropriate to solve a particular social problem?
- Was there convergent evolution in the apportionment of the levers of reciprocity in societies facing the same ecological conditions?
- Can artificial intelligence and in particular ‘human–machine teaming’ help to design inter-temporal incentives solving specific social problems?

Finally, our analysis raises the following broad question: can intertemporal incentives be systematically designed to favour global welfare issues? Global planetary cooperation tends to be ruled out in accounts of human sociality based on cultural group selection processes, since cooperation at a planetary scale then necessarily relies on competition between planets to obtain. In contrast, nothing in the reciprocity principle prevents interactions within and between groups to build up intertemporal incentives targeted to meet global welfare outcomes. Answering this question and thinking about possible incentive design pathways to planet-wide cooperation for specific action situations should be useful to improve the human capacity to engineer solutions to global societal problems, from environmental change and resource use to the control of warbots and the human genetic load (see also Stewart, 2020). While pushing the cooperation frontier will not be easy, since it requires the finding of suitable combinations of the levers of reciprocity working for billions of different people, the potential unlimited plasticity and scalability of the reciprocity principle allow us to keep the faith in the human race.

## Data Availability

There are no data to report.

## Supplementary material

For supplementary material accompanying this paper visit https://doi.org/10.1017/ehs.2022.7. click here to view supplementary material
