# Supplementary material for: Four levers of reciprocity across human societies: concepts, analysis and predictions
Source: Evol Hum Sci. 2022 Feb 21;4:e11. doi: 10.1017/ehs.2022.7 (PMC10426116; doi:10.1017/ehs.2022.7)
Supplement: Supplementary file 1 [file S2513843X2200007Xsup001.zip › SuppMat.pdf]

Supplementary Material for "Four levers of reciprocity across human societies: concepts, analysis and predictions"

by Laurent Lehmann, Simon T. Powers and Carel P. van Schaik

Here, we present the structural dimension for the five societal types in five distinct tables. These tables are structured according to our game theoretic framework (recall section 2.3 of the main text). The tables were constructed using classic syntheses, like Johnson and Earle (1987) and Maryanski and Turner (1993), and validated and complemented with recent textbooks on big-history (Christian et al., 2014) and macrosociology (Nolan and Lenski, 2014), as well as political science literature (all references used are listed in the tables' footnotes).

Table 1: Mobile foragers

Dimension	Description
**Population**	
Density	Range between 0.005-0.9 individuals per km^2.
Spatial	Local groups (bands) of small size; 20-100 individuals forming a few families living in non-permanent settlements sometimes with temporary household shelters but relocated at least seasonally or depending on conditions. Bands are usually embedded within a network of similar bands, forming communities up to a few thousand individuals that define the scale of society.
Interactions	Mainly among band members, occasionally with individuals from other bands.
**Economy**	
Subsistence	Communal hunting of animals and gathering of plants.
Production	Clear division of labour between men hunting more unpredictable and dangerous resources, and females gathering more dependable yield. Use of hunting and cooking equipment but infrastructure for resource production essentially non-existent; occasionally known to burn areas and dig canals.
Distribution	Tendency of private ownership of gathered resources but communal ownership of hunted resources. Hence, resources are widely shared at the local group level. No surplus, hence no material inequality nor much trade except with members from other bands or communities usually under the form of gifts that are reciprocated later.
Appropriation	Since no surplus and food stores, little to appropriate from conspecifics and thus to protect, except for females and perhaps fire and recognized communal ownership of land or waterholes. Occasional contests and raids between groups, more rarely battles, but no defensive infrastructures.
**Polity**	
Decision-making	Decentralized consensus building at the band level and possibly at the community level.
Power	Equal authority by all group members. No delegation of power, except to delegated speakers when decisions are taken at the community level. Hence, no central authority or leadership.
Rules	Informal customs and norms.
Enforcement	Decentralized as well as centralized at the group level through consensus based group-level decisions that enable coalitionary power. Penalties are mainly reputational or due to ostracism, but sometimes killing can occur.

References: Service (1975); Johnson and Earle (1987); Maryanski and Turner (1993); Christian (2004); Kaplan et al. (2005); Gat (2006); Kaplan et al. (2009); Fukuyama (2011); Gualla (2012); Christian et al. (2014); Nolan and Lenski (2014); van Schaik (2016); Turner and Maryanski (2016); Gat (2017); Smil (2017); Stutz (2020).

Table 2: Horticulturalists

Dimension	Description
**Population**	
Density	Range between 10-60 individuals per km^2.
Spatial	Local groups of small size; 20-200 individuals. Local groups are partly sedentary and form small villages where individuals live in agglutinated semi-permanent communal houses or longhouses (usually one or very few at the local level). Local groups are usually embedded into larger communities or tribes that can reach around 5,000 individuals. The tribal structure can give rise to chiefdoms in areas of high resource productivity.
Interactions	Mainly among local group members, occasionally with individuals from other groups.
**Economy**	
Subsistence	Hunting but mainly semi-permanent communal food planting and harvesting (extremely variable from fruits to roots and tubers), and tending of small domestic animals such as goats, sheep, chickens.
Production	Division of labour between females doing mainly gardening and males doing more hunting, tool/weapon-making, house-building, and boat-building. Use of equipment and tools for production as well as some infrastructures of production, such as field clearing (sometimes fire-assisted), paths clearing, developing irrigation systems.
Distribution	Generally low productivity and no private resource ownership and redistribution of produced resources at the group level. Tendency to some long distance trade by men. Sometimes surplus with appearance of communally owned food stores and in chiefdoms private ownership of such food stores.
Appropriation	In ecologies with more densities and/or production, presence of fighting and war raids above a certain density, which results in food and bride capture. But no clear infrastructure for appropriation nor defense.
**Polity**	
Decision-making	Variable at the village level. Mix between decentralized consensus building and centralized leadership generally organized along kinship lines. In chiefdoms, there is more hierarchy and thus more centralization of decision-making.
Power	In principle, equal authority by all group members but some delegation of power to the leadership.
Rules	Informal customs and norms. Sometimes differentiation between *substantive law*–rules describing how members of a society are to behave–and *procedural law*–rules describing procedures for developing and enforcing the substantive law itself, yet all legal codes remain unwritten and not clearly codified.
Enforcement	Decentralized as well as centralized at the group level. Leadership has staff and sometimes small armies. This results in the slow emergence of coercive capacity by a segment of society.

References: Service (1975); Johnson and Earle (1987); Maryanski and Turner (1993); Earle (1997); Christian (2004); Kaplan et al. (2005); Gat (2006); Kaplan et al. (2009); Fukuyama (2011); Kelly (2013); Christian et al. (2014); Nolan and Lenski (2014); Flannery and Marcus (2014); Turner and Maryanski (2016); Gat (2017); Smil (2017).

Table 3: Pre-state agriculturalists

Dimension	Description
**Population**	
Density	Average at least 40 individuals per km^2.
Spatial	Local groups of highly variable size, could consist of single families but often consists of more or less scattered settlements consisting of several permanent separate households (villages). Local groups are always hierarchically embedded in larger units such as tribes and chiefdoms, with perhaps 10,000 members but still based on a single ethnic unit with one language.
Interactions	Mainly through flat networks of exchange at the local group level.
**Economy**	
Subsistence	Intensive cultivation of variable scale, usually involving metal tools and draft animals, which generates tradeable and taxable surplus.
Production	Division of labour between male and female with increased occupational specialization into farming, house-building, metal-working, weaving, pottery-making and commerce. Appearance of sizeable infrastructure for resource production, such as roads for vehicles, land improvement including deforestation, irrigation systems and canals, stores of grain and large villages.
Distribution	Largely private ownership of resources, land and real estate (except communal grazing areas), which results in varying degree of material inequality. Redistribution of resources as a result of flourishing exchange, which results in some long-distance trade networks largely between societies and largely about non-essential luxury goods.
Appropriation	Owing to increased density and productivity warfare (or raiding) is prevalent among localities and created needs for communal defence. Appearance of infrastructure of protection such as walled villages.
**Polity**	
Decision-making	Variable but tends to be centralized. At the village level explicit leaders taking decisions in consultations with others. Appearance of leadership at the community level.
Power	Targeted delegation of power towards the local or regional leadership. Authority starts to be concentrated in the leadership in chiefdoms.
Rules	Customs and norms codified by tradition. Differentiation between substantive and procedural law.
Enforcement	Leaders gain centralised coercive capacity allowing for the extraction of labour but there is no monopoly on coercion.

References: Service (1975); Johnson and Earle (1987); Maryanski and Turner (1993); Earle (1997); Christian (2004); Gat (2006); Fukuyama (2011); Christian et al. (2014); Nolan and Lenski (2014); Flannery and Marcus (2014); Turner and Maryanski (2016); Scott (2017). In Tables 3-5, we use the notion of flat and hierarchical networks, meaning they have many hubs following Christian (2004, p. 292). In Tables 3-5, we also follow Brousseau et al. (2010, p. 258) in using the notion of *targeted delegation* of power, which consists of granting a limited amount of focused authority, as well as *extended delegation* of power, which consists of delegating most decisions to the central authority.

Table 4: State-based agriculturalists (archaic states)

Dimension	Description
**Population**	
Density	Average well over 100 individuals per km^2.
Spatial	Local groups range from villages of various sizes up to cities, which are multi-ethnic and based on the fusion of communities or sometimes massive import of people of different languages. Hierarchical networks of villages and cities form *states*, which is a set of people united into a single territorial limit with a central government. (The defining feature of the state is the monopoly of coercion over society members and the ability to tax).
Interactions	Through various local exchange networks that become hierarchical and sometimes extend to the the societal level.
**Economy**	
Subsistence	Large-scale intensive permanent cultivation generating large surplus where one farmer can sustain several non-farmers specialists.
Production	Continuing increased of division of labour with appearance of new occupational trades, such as soldiers and bureaucrats; slaves, serfs and elites; and regional specializations. Continuing increase of infrastructure for food production such as roads, canals, large scale irrigation systems, large stores, walled cities with infrastructures for waste management, and regional integration.
Distribution	In principle private ownership of resources, yet production is heavily taxed and often centrally controlled. Trade within societies as well as between societies important and involved importation of metal and building materials.
Appropriation	Cities are defended and there are incessant conflicts, often armed, between states having developed military infrastructure.
**Polity**	
Decision-making	Fully centralized in an hereditary monarchy that is supported by elites and subtented by a hierarchy of officials.
Power	Extended delegation of powers towards the leadership, which retains unequal authority and tends towards the despotic. The power structure within society is very hierarchical.
Rules	Beginning of written legal rules. Clear differentiation between procedural and substantive law.
Enforcement	Organized state bureaucracy, consisting of religious elites and military and administrative personal, including slaves. Bureaucracy can enforce taxation and essentially everything else by the use of the monopoly of force.

References: Service (1975); Johnson and Earle (1987); Maryanski and Turner (1993); Earle (1997); Christian (2004); Gat (2006); Fukuyama (2011); Christian et al. (2014); Nolan and Lenski (2014); Hoffman (2015); Turner and Maryanski (2016); Gat (2017); Scott (2017); Smil (2017).

Table 5: Liberal democracies

Dimension	Description
**Population**	
Density	Up to 2000 individuals per km^2.
Spatial	Variable local group size, ranging from small villages to mega-cities where millions of families reside. These common residence groups are embedded in nation-states of highly variable size.
Interactions	In various hierarchical networks pertaining to work and leisure and where these networks can be world wide.
**Economy**	
Subsistence	Large-scale intensive permanent mechanized cultivation and industrial food and technological production allowing for massive surplus per farmer (one American farmer now produces enough food to feed more than 150 people).
Production	Decrease of division of labour between males and females yet increase of overall division of labour and specialization with strong reliance on machines and computerized information-processing mechanisms shifting work increasingly to secondary and tertiary industries. Extensive infrastructure for food and good production such as roads, water canals, various forms of energy and information networks. Very high level of organization of cities, sanitation, organization of schools, and overall civil society infrastructures. Virtually all economic activity is carried out not by individuals but by organizations.
Distribution	Private property of resources yet large inequality in resource ownership. Resources or transformation thereof (goods) are exchanged on decentralized markets that cause an endless transformation of materials, products, energy, and information. Global networks of exchange connecting the whole world. Resources under the form of wealth are also partly redistributed by the state by way of levying taxes.
Appropriation	Conscripted armies and high-tech military infrastructure often leading technological innovation that later benefit civil society. Yet less warfare between nation (liberal democracies themselves do not wage war against each other).
**Polity**	
Decision-making	Centralized in the hands of a mix of elected leaders and legislative bodies.
Power	Nominal equality in power, but extended delegation of powers towards the centralized government ("constitutional delegation of power"). The rule of law entails that the government is subject to disclose legal codes and processes resulting in check-and-balances feedback between civil society and the government. The power structure within society is thus relatively flat ("universal rights to anybody"), yet can be concentrated in influence groups.
Rules	Large bodies of written procedural and substantive law regulating essentially all spheres of behaviour. Legal protection of individuals, corporations, select assets, and parts of the environment.
Enforcement	Large standing military, police, and bureaucratic systems. These high technology systems guarantee the monopoly on force to the state and enable taxation and redistribution, enforcement of rules and rights, and adjudicating disputes and fostering scientific research.

References: Johnson and Earle (1987); Maryanski and Turner (1993); Christian (2004); Mankiv (2010); Fukuyama (2011, 2014); Christian et al. (2014); Nolan and Lenski (2014); Hoffman (2015); Gat (2017); Turner and Maryanski (2016); Smil (2017); Pastor (2019)

References

Brousseau, E., Y. Schemeil, and J. Sgard. 2010. Bargaining on law and bureaucracies: A constitutional theory of development. *Journal of Comparative Economics* pp. 253–266.

Christian, D. 2004. Maps of Time: An Introduction to Big History. University of California Press, Berkeley, CA.

Christian, D., C. Stokes Brown, and C. Benjamin. 2014. Big History: Between Nothing and Everything. McGraw-Hill, New York.

Earle, T. 1997. How Chiefs Come to Power: The Political Economy in Prehistory. Stanford University Press, Stanford, CA.

Flannery, K. and J. Marcus. 2014. The Creation of Inequality. Harvard University Press, Harvard.

Fukuyama, F. 2011. The Origins of Political Order. Profile Books, London, UK.

Fukuyama, F. 2014. Political Order and Political Decay. Farrar, Strauss and Giroux, New York.

Gat, A. 2006. War in Human Civilization. Oxford University Press, Oxford.

Gat, A. 2017. The Causes of War and the Spread of Peace: But Will War Rebound? Oxford University Press, Oxford.

Gualla, F. 2012. Reciprocity: weak or strong? What punishment experiments do (and do not) demonstrate. *Behavioral and Brain Sciences* 35:1–59.

Hoffman, P. T. 2015. What do states do? Politics and history. *The Journal of Economic History* 75:303–332.

Johnson, A. W. and T. Earle. 1987. The Evolution of Human Societies. Stanford University Press.

Kaplan, H., M. Gurven, K. Hill, and A. M. Hurtado. 2005. The natural history of human food sharing and cooperation: A review and a new multi–individual approach to the negotiation of norms. In Gintis, H., S. Bowles, R. Boyd, and E. Fehr (eds.), *Moral sentiments and Material Interests: The Foundations of Cooperation in Economic Life*, pp. 75–113. MIT Press, Cambridge, MA.

Kaplan, H., P. L. Hooper, and M. Gurven. 2009. The evolutionary and ecological roots of human social organization. *Philosophical Transactions of the Royal Society B-Biological Sciences* 364:3289–3299.

Kelly, R. L. 2013. The lifeways of hunter-gatherers: The foraging spectrum. Cambridge University Press, Cambridge, UK, second edn.

Mankiv, G. N. 2010. Brief Principles of Macroeconomics. South-Western, sixth edition edn.

Maryanski, A. and H. Turner. 1993. The Social Cage: Human Nature and the Evolution of Society. Stanford University Press, Stanford, CA.

Nolan, P. and G. Lenski. 2014. Human Societies: An Introduction to Macrosociology. Paradigm Publishers, London, 11th edn.

Pastor, K. 2019. The Code of Capital: How the Law Creates Wealth and Inequality. Princetion University Press, Princeton, NJ.

Scott, J. 2017. Against the Grain: a Deep History of the Earliest States. Yale University Press, New Haven.

Service, E. R. 1975. Origins of the State and Civilization: The Process of Cultural Evolution. W. W. Norton and Company, New York.

Smil, V. 2017. Energy and Civilization: a History. The MIT Press, Cambridge, MA.

Stutz, A. J. 2020. A niche of their own: population dynamics, niche diversification, and biopolitics in the recent biocultural evolution of hunter-gatherers. *Journal of Anthropological Archaeology* p. 101120.

Turner, J. H. and A. Maryanski. 2016. On the Origin of Societies by Natural Selection. Routledge, New York.

van Schaik, C. P. 2016. The Primate Origin of Human Behavior. Wiley-Blackwell, New Jersey.
